# Application of lactose based autoinduction for heterologous production of an active [NiFe] hydrogenase in *E. coli*

**DOI:** 10.1007/s10529-025-03594-4

**Published:** 2025-05-05

**Authors:** Francisco de la Fuente-Kratzborn, Qin Fan, Peter Neubauer, Matthias Gimpel

**Affiliations:** Chair of Bioprocess Engineering, Institute of Biotechnology, Technische Universität Berlin, Ackerstr. 76, ACK24, D‑13355 Berlin, Germany

**Keywords:** [NiFe]-hydrogenase, Autoinduction, Regulatory hydrogenase, *Cupriavidus necator*

## Abstract

**Objectives:**

This study aims to assess whether a fed-batch-based auto-induction method can enhance active hydrogenase production, encompassing cofactor formation and space–time yield.

**Results:**

The recombinant *Escherichia coli* strain BQF8RH8, possessing plasmids for assembly and proper maturation of *Cupriavidus necator* regulatory hydrogenase (RH), was cultivated in the fed-batch like EnPresso B medium with an autoinduction lactose-based system. In contrast to classical IPTG induction previously performed, we obtained active RH demonstrating the feasibility of the process for active hydrogenase production.

**Conclusion:**

Our results affirm the viability of the previously developed auto-induction strategy also for a functional hydrogenase with the complex maturation process. This significantly accelerates the process and enhances hydrogenase productivity.

## Introduction

[NiFe] Hydrogenases are metalloenzymes that hold significant promise as biocatalysts e.g. as part of bioanodes in biofuel cells, or in H_2_-facilitated cofactor recycling systems (Mertens and Liese 2004; Chenevier et al. 2013; Reeve et al. 2017). Despite the important achievements in the production and understanding of these promising but complex enzymes (Lubitz et al. 2014; Ogata et al. 2016; Ogo et al. 2020), the heterologous production of hydrogenases remains challenging. Besides the elevated cost of enzyme synthesis, hydrogenases are quite sensitive to ambient O_2_ and CO, which in turn restricts their use in industrial applications (Wulff et al. 2014; Sokolova and Vincent 2024). So far, heterologous production of [NiFe] hydrogenases was only achieved at lab scale and in a limited number of bacterial species including *E. coli, Desulfovibrio fructovorans* or *Ralstonia eutropha* (rev. Fan et al. 2020). Although the yields obtained heterologously considerably exceed the yields from the natural producer, they are in the low range of mg g^−1^ or mg L^−1^ (Fan et al. 2020), highlighting the challenges in heterologous hydrogenase production.

To make hydrogenase more accessible, we recently succeeded in developing a strategy for the functional heterologous production of the oxygen-tolerant regulatory hydrogenase (RH) from *Cupriavidus necator* (also known as *Ralstonia eutropha*) in *E. coli*. With our strategy, we obtained significantly higher yields compared to the native host, with about 250 mg L^−1^ of apoenzyme (~ 0.1 mg g^−1^ h^−1^) (Fan et al. 2021a). The implementation of a fed-batch-like process using the EnBase® strategy not only led to a significant increase in biomass but also to an increase in volumetric and specific RH yield (Fan et al. 2021a). This system is based on a standard *E. coli* mineral salt medium supplemented with a non-metabolizable polymer as a carbon source and a biocatalytic glucose release (Krause et al. 2016). The slow and controlled glucose release from a polymer allows cells to reach high cell densities (OD_600_ of 20–50) and achieves improved protein yields in recombinant production. This cultivation system has demonstrated considerable success, with increased robustness, reproducibility, and reduced acidifying side products compared to standard batch systems in shake flasks. Additionally, its enzyme-mediated glucose release supports stable pH levels and linear growth rates, further enhancing protein production efficiency (Li et al. 2014, 2015; Ukkonen et al. 2017).

The subsequent improvement of the production process, in particular the supplementation with nickel and iron ions and the co-expression of the native maturation genes, led to the production of an active RH (0.6 U mg^−1^; 80 mg L^−1^; ~ 0.02 mg g^−1^ h^−1^) (Fan et al. 2022a). Moreover, with extended production time, a specific activity comparable to those of the native enzyme could be achieved, however, at the expense of the production yield (3 U mg^−1^; 40 mg L^−1^; ~ 0.008 mg g^−1^ h^−1^) (Fan et al. 2022a). By conducting glucose-limited high cell density fed-batch cultivation in benchtop bioreactors under the optimized conditions derived from shake flask cultures, more than 100-fold increase in soluble active RH production has been achieved, alongside a comparable specific activity (1.3 U mg^−1^; > 130 mg L^−1^; ~ 0.03 mg g^−1^ h^−1^), compared to RH yields obtained in other laboratory bioreactor scale processes (Bernhard et al. 2001; Fan et al. 2022b).

In *E. coli* expression of recombinant protein encoding genes is typically controlled by lactose-inducible promoter systems (Rosano and Ceccarelli 2014). Autoinduction with lactose as inducer is a straightforward alternative to manually triggering gene expression from P_lac_-derived promoters with IPTG. This method initiates recombinant protein synthesis by leveraging glucose catabolite repression and inducer exclusion principles, which are activated in the presence of glucose (Fischer et al. 1998; Stülke and Hillen 1999; Bettenbrock et al. 2006). When glucose levels are consumed, lactose is taken up by the cell, leading to the induction of the Lac promoter (Görke and Stülke 2008). Lactose autoinduction media have been found to result in higher cell densities and greater yields of target proteins compared to manual IPTG induction in commonly used media (Neubauer et al. 1991; Neubauer et al. 1992; Studier 2005; Gordon et al. 2008). Additionally, cells can be added directly into autoinducing medium and allow them to grow to saturation without the requirement of monitoring their growth or administration of the inducer at precise intervals, providing notable advantages for high-throughput applications and the organization of shake flask experiments. Recently, lactose autoinduction was successfully applied for heterologous RH production in a fed-batch like EnPressoB process (Fan et al. 2021b). Here, lactose autoinduction remarkably enhanced volumetric (280 mg L^−1^) and specific yield (7.5 mg g^−1^) while reducing the process duration (Fan et al. 2021b). However, the suitability of autoinduction was only demonstrated for strain BQF8RH, lacking the maturation genes, thus producing an inactive RH.

In the present study, we aimed at investigating whether lactose autoinduction is also suitable to produce active RH. We used strain BQF8RH8 and performed cultivations under the previously optimized conditions.

## Materials and methods

### Bacterial strain and culture medium

For this study, we utilized the *Escherichia coli* strain BQF8RH8, which contains the plasmids pQF8 and pQF18 (Fan et al. 2022a), as the host for production. The pQF8 plasmid carries genes encoding the RH structural subunits, under control of P_lac_ promoter derivative and plasmid pQF18 harbours the genes encoding proteins for RH maturation (HypA1B1 F1 CDEX) along with the nickel permease HoxN, both controlled by a P_tac_ promoter (Fan et al. 2022a).

Pre-cultures were cultivated in LB medium, composed of 10 g/L tryptone, 5 g/L yeast extract, and 5 g/L NaCl. For the main cultures, we employed EnPresso B medium, a typical *E. coli* mineral salt medium supplemented with a non-metabolizable glucose polymer and an enzyme for controlled glucose release (Soini et al. 2008). The EnPresso B medium was used according to the manufacturer’s instructions (EnPresso GmbH, Germany).

### Cultivation conditions

Pre-cultures were prepared as follows: A single *E. coli* colony was introduced into 10 mL LB broth supplemented with chloramphenicol (34 μg/mL) and kanamycin (50 μg/mL). The culture was incubated at 37 °C for 7 h with agitation at 250 rpm (Infors HT, 25 mm orbit, Switzerland).

For the main culture, cells were transferred into 50 mL of EnPresso B medium at an initial OD_600_ of 0.2, using 250 mL baffled shake flasks (PreSens, Germany). Cultivation parameters, including dissolved oxygen (DO) and pH, were continuously tracked with an SFR shake flask reader in a Kuhner LTX orbital shaker (50 mm offset, Adolf Kühner AG, Switzerland).

The medium was supplemented with selective antibiotics and 1 × Booster tablet and 3 U*L^−1^ following the supplier’s recommendations (EnPresso GmbH, Germany). Expression of RH was triggered by addition of 2 g/L lactose and 0.25 g/L glucose, following Fan et al. (2021b). To ensure proper formation of the NiFe(CN)_2_CO cofactor, 0.1 mM NiSO_4_ and 0.1 mM FeCl_3_ were added at the start of the cultivation.

Cultures were incubated at 18 °C for 24, 48, and 96 h, respectively. At designated intervals, samples were collected to determine cell growth (OD_600_), glucose levels and RH production. Cells were harvested via centrifugation (8000 × g, 10 min, 4 °C) and pellets stored until further use at − 20 °C.

### Measurement of glucose

To assess glucose levels, samples were adjusted to an OD_600_ of 10 before being subjected to centrifugation at 21,500 × g for 10 min at 4 °C. The resulting supernatants were stored at − 20 °C until analysis. Glucose concentration was quantified using a Cedex Bio HT Analyzer (Roche Diagnostics International AG, Switzerland) in combination with the Glucose Bio HT test kit, strictly according to the manufacturer’s protocol.

### Determination of total RH yield

For total protein analysis, cell pellets (adjusted to an OD_600_ of 2.5) were resuspended in 300 µL of 8 M urea. The samples were then combined in a 1:1 ratio with 2 × SDS sample buffer and heated at 98 °C for 10 min. Following centrifugation, 15 μL of each denatured sample was loaded onto 12% PAA gels for SDS-PAGE, followed by western blotting.

Western blotting was carried out as previously described (Fan et al. 2021a). In brief, proteins were transferred onto a 0.45 μm PVDF membrane (Carl Roth, Germany) using the Transblot Turbo Transfer system (Bio-Rad, Germany) at 1.3 A and 25 V for 30 min. Detection of Strep-tagged HoxB was achieved using an anti-Strep-tag®II mouse antibody (IBA GmbH, Germany) at a 1:2500 dilution (0.25 μg/mL), followed by incubation with an alkaline phosphatase (AP)-conjugated goat anti-mouse IgG secondary antibody (1:8000 dilution, Sigma-Aldrich, Germany). Bands were visualized via BCIP/NBT substrate reaction, as detailed by Fan et al. (2021a).

Blots were digitized using a ChemiDoc imaging system (Bio-Rad, Germany) and analyzed with ImageJ. RH concentrations were quantified by comparing band intensities against a reference standard of purified Strep-tagged HoxC.

### RH purification and activity assay

Cell pellets were resuspended in washing buffer A (100 mM Tris–HCl, pH 8.0, 150 mM NaCl) at a ratio of 10 mL per gram of wet biomass, with the addition of 12 µL/mL PMSF. Cell disruption was carried out by sonication using a 7 mm sonotrode at 30% amplitude in alternating 30 s on/off cycles for a total duration of 10 min. The crude lysate was then centrifuged at 14,000 × rpm for 15 min at 4 °C to separate the soluble protein fraction.

Clarified lysates were loaded onto Strep-Tactin® Gravity Superflow® columns (IBA GmbH, Germany) with a 250 µL bed volume and purified according to the manufacturer’s protocol. The isolated proteins were analyzed via SDS-PAGE, and band intensities were quantified using ImageJ, as previously outlined (Fan et al. 2021a, b).

To evaluate RH enzymatic activity, hydrogen oxidation was measured spectrophotometrically using a Cary50 UV/Vis spectrophotometer (Varian, Agilent, Santa Clara, California). The H₂ uptake assay employed methylene blue as the electron acceptor, following the methodology described by Lenz et al. (2018).

All experiments were conducted with three biological replicates, and standard deviations were derived from at least two independent technical replicates.

## Results and discussion

### RH production using lactose-based autoinduction

The suitability of autoinduction for heterologous hydrogenase production in a fed-batch like cultivation was proven recently using strain BQF8RH. The RH yields obtained with lactose autoinduction (280 mg L^−1^) were remarkably higher compared to classical IPTG induction (130 mg L^−1^) (Fan et al. 2021b), however the produced RH was inactive due to the absence of the [NiFe]-cofactor. In contrast active RH (40 mg L^−1^ with 3 U mg^−1^) could be obtained from the improved strain BQF8RH8 (Fan et al. 2022a), coexpressing the RH maturation genes. Besides co-expression of the maturation genes optimization of the process conditions was required for production of an active RH (Fan et al. 2022a). To test whether autoinduction is also suitable to produce active RH, we carried out shake flask cultivations in EnPresso medium with *E. coli* BQF8RH8.

**Fig. 1:**
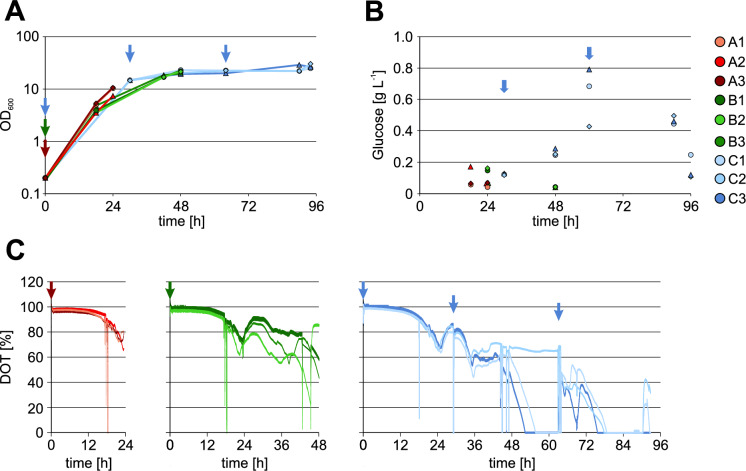
Cultivation of *E. coli* BQF8RH8. Cells were cultivated in UYFs with EnPresso B medium. For autoinduction controlled RH synthesis 2 g L^−1^ lactose were added from the beginning of the cultivation, while 0.5 g L^−1^ glucose were added to prevent premature RH production. Cultures were grown for 24 h (red), 48 h (green) or 96 h (blue). Arrows indicate booster additions **A** Growth curve measured as OD_600_. **B** Glucose concentrations measured using Cedex Bio HT analyzer at the established intervals. **C** DO profile. The cultivations were performed as independent biological triplicates

The growth was determined at established intervals by measuring the OD_600_ of each flask. As prolonged cultivation was shown to improve RH activity while shorter cultivation times resulted in higher yields (Fan et al. 2022a) cultures were harvested after 24 h, 48 h and 96 h of cultivation, respectively. During the first 24 h, the glucose concentration remains non-limiting due to both the initial glucose supply and the sustained release of glucose from the EnPresso medium. This facilitates rapid cell growth, as evidenced by the sharp decrease in dissolved oxygen (DO) levels (Fig. 1C). As the carbon source becomes limited, the culture transitions to the stationary growth phase, leading to an increase in DO, indicative of reduced metabolic activity. Following adaptation to carbon limitation, residual metabolic processes continue, resulting in oxygen consumption and a subsequent gradual decline in DO levels. This is corroborated by the immediate drop in DO following the addition of a booster at 30 h suggesting free capacity for nutrient uptake. After 60 h, the complete depletion of the carbon source is marked by a pronounced rise in DO, followed by a temporary decrease upon further booster addition. Nevertheless, the OD levels remain stable after 30 h despite continued booster addition to the 96 h cultures (Fig. 1A). Thus, it can be assumed that the additional booster does not accelerate cell growth but contributes to maintenance of cell physiology and protein production. As expected, the glucose levels remain low over the entire cultivation and increase only after booster tablet addition (Fig. 1B) and thus should allow lactose-based autoinduction.

**Fig. 2:**
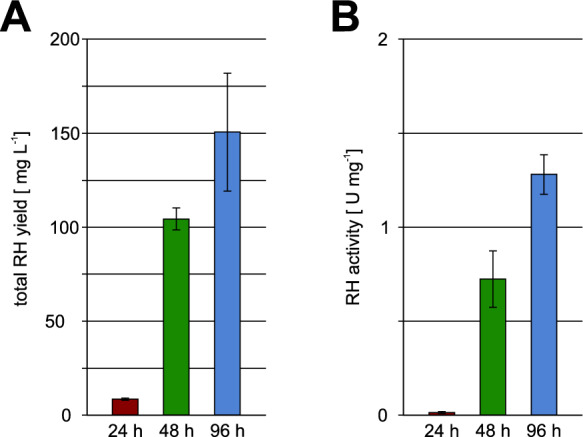
Determination of RH yield and activity. **A** Total RH yield, obtained from *E. coli* BQF8RH8 cultivated under autoinduction conditions in EnPresso B medium, was calculated from Western blots as described in Materials and Methods. **B** Specific RH activity was determined as previously described (Fan et al 2022a)

However, after 24 h of cultivation, the RH yield was considerably low (Fig. 2A). The low RH yield contrasts with the previous results using strain BQF8RH (Fan et al. 2021b) where about 280 mg L^−1^ could be obtained already after 24 h of cultivation. A major difference between both processes is the production temperature. While 30 °C were used for the previous process with strain BQF8RH (Fan et al. 2021b), 18 °C were used here instead. The reduced temperature results in slower cell growth and thus might reduce glucose uptake and consumption leading to higher residual glucose levels during the first 24 h. Consequently, the presence of glucose prevents RH expression during the initial growth phase leading to the observed low RH yields. This demonstrates the need for sufficient time to consume the enzymatically released glucose from the polymer and for autoinduction to start, followed by subsequent protein production. In contrast, prolonged cultivation resulted in a significant increase of total RH yield reaching about 100 mg L^−1^ after 48 h and around 150 mg L^−1^ after 96 h, respectively (Fig. 2A). As expected, the soluble RH yields exceed the volumetric yields obtained with classical IPTG induction by 2- to 4-fold (Fan et al. 2022a), demonstrating the suitability of lactose autoinduction for RH production with strain BQF8RH8. However, the 300 mg L^−1^ obtained with strain BQF8RH (Fan et al. 2021b) could not be reached. This may be due to different production conditions.

The lower temperature decreases the growth rate as well as the protein production. Compared to the previous results (Fan et al. 2021b), about 2-fold lower ODs were obtained which significantly affects the volumetric RH yield. On the other hand, strain BQF8RH did not contain plasmid pQF18 encoding the RH maturation genes. While the presence of the maturation genes is crucial for RH activity, it is also a metabolic burden on the cells, reducing the capacity for recombinant protein production and thus the final product yield. A similar effect on RH yield was also observed for production with IPTG induction (Fan et al. 2021a, 2022a). Interestingly, the RH yield steadily increased over the cultivation time. This contrasts with the previous observation that prolonged cultivation results in a decrease in the volumetric yield (Fan et al. 2022a) and might be attributed to the additional booster addition. This additional booster provides nutrients that can be used to maintain cell physiology and thus prevent product degradation as observed before (Fan et al. 2022a).

### Production of active RH with lactose autoinduction is possible

The production of enzymatically active protein is essential for a bioprocess to be considered successful. Thus, we measured the H_2_-oxidation activity of the produced RH after 24 h, 48 h and 96 h of cultivation with lactose autoinduction. While no activity was detectable in RH samples purified after 24 h, the RH samples purified after 48 h showed a specific activity of about 0.7 U mg^−1^ that further increased to > 1.3 U mg^−1^ in the 96 h samples (Fig. 2B). As nearly no RH protein could be obtained after 24 h the lack of activity is not surprising. The maturation genes on plasmid pQF18 are under control of the P_tac_ promoter and thus susceptible to glucose. Even though expression of structural and maturation genes starts after about 20 h when the initial glucose is exhausted (Fig. 1C), the maturation process requires additional time and cannot be completed. The increase in specific activity over time observed here (Fig. 2B) and reported previously (Fan et al. 2022a, b) supports this assumption. Although the maximum activity of 3.0 U mg^−1^ achieved with IPTG induction (Fan et al. 2022a) has not yet been reached, it is noteworthy that this lactose-based autoinduction allows controlled RH expression, resulting in production of an RH with reasonable activity, but at almost fourfold higher yield compared to the previously reported process with IPTG induction (Fan et al. 2022a).

## Conclusion

In this study, we used the improved strain BQF8RH8, which produces catalytically active RH with activities comparable to the RH purified from *C. necator* and applied lactose-based autoinduction using EnPresso B medium. Although the RH yields did not reach the optimized levels for the strain BQF8RH (Fan et al. 2021b), they are higher than those previously achieved with the strain BQF8RH8 (Fan et al. 2022a). Furthermore, the RH exhibited enzymatic activity, demonstrating the effectiveness of autoinduction for the heterologous production of an active hydrogenase. This serves as a basis for future improvement of the autoinduction-based processes and scale-up of the production in bioreactors.

## Data Availability

The datasets generated during and/or analysed during the current study are available in the DepositOnce repository, https://doi.org/10.14279/depositonce-22978.
